# Host Cell Poly(ADP-Ribose) Glycohydrolase Is Crucial for *Trypanosoma cruzi* Infection Cycle

**DOI:** 10.1371/journal.pone.0067356

**Published:** 2013-06-12

**Authors:** Salomé C. Vilchez Larrea, Mariana Schlesinger, María L. Kevorkian, Mirtha M. Flawiá, Guillermo D. Alonso, Silvia H. Fernández Villamil

**Affiliations:** 1 Instituto de Investigaciones en Ingeniería Genética y Biología Molecular “Dr. Héctor N. Torres”, Consejo Nacional de Investigaciones Científicas y Técnicas, Ciudad Autónoma de Buenos Aires, Argentina.; 2 Departamento de Fisiología, Biología Molecular y Celular, Facultad de Ciencias Exactas y Naturales, Universidad de Buenos Aires, Ciudad Autónoma de Buenos Aires, Argentina; 3 Departamento de Química Biológica, Facultad de Farmacia y Bioquímica, Universidad de Buenos Aires, Ciudad Autónoma de Buenos Aires, Argentina; Federal University of São Paulo, Brazil

## Abstract

*Trypanosoma cruzi*, etiological agent of Chagas’ disease, has a complex life cycle which involves the invasion of mammalian host cells, differentiation and intracellular replication. Here we report the first insights into the biological role of a poly(ADP-ribose) glycohydrolase in a trypanosomatid (TcPARG). *In silico* analysis of the TcPARG gene pointed out the conservation of key residues involved in the catalytic process and, by Western blot, we demonstrated that it is expressed in a life stage-dependant manner. Indirect immunofluorescence assays and electron microscopy using an anti-TcPARG antibody showed that this enzyme is localized in the nucleus independently of the presence of DNA damage or cell cycle stage. The addition of poly(ADP-ribose) glycohydrolase inhibitors ADP-HPD (adenosine diphosphate (hydroxymethyl) pyrrolidinediol) or DEA (6,9-diamino-2-ethoxyacridine lactate monohydrate) to the culture media, both at a 1 µM concentration, reduced *in vitro* epimastigote growth by 35% and 37% respectively, when compared to control cultures. We also showed that ADP-HPD 1 µM can lead to an alteration in the progression of the cell cycle in hydroxyurea synchronized cultures of *T. cruzi* epimastigotes. Outstandingly, here we demonstrate that the lack of poly(ADP-ribose) glycohydrolase activity in Vero and A549 host cells, achieved by chemical inhibition or iRNA, produces the reduction of the percentage of infected cells as well as the number of amastigotes per cell and trypomastigotes released, leading to a nearly complete abrogation of the infection process. We conclude that both, *T. cruzi* and the host, poly(ADP-ribose) glycohydrolase activities are important players in the life cycle of *Trypanosoma cruzi*, emerging as a promising therapeutic target for the treatment of Chagas’ disease.

## Introduction

Poly-ADP-ribose (pADPr) signaling is common to various nuclear processes related to DNA metabolism. As a reversible modification of many nuclear proteins, it is regulated by a delicate balance of synthesis and degradation. Poly(ADP-ribosyl) ation is an early cellular response to DNA damage and is a concerted and dynamic process: poly(ADP-ribose) polymerases (PARPs) catalyze the transfer of ADP-ribose (ADPr) and attach them to specific target proteins, whereas poly(ADP-ribose) glycohydrolase (PARG) represents the main pADPr hydrolyzing activity in the cell to ADPr units. Poly(ADP-ribose) chains are thus transient, and it is suggested that once other proteins have localized to the damage site, pADPr must be removed before repair can take place. Poly(ADP-ribose) glycohydrolase (PARG) is the endo-

exoglycohydrolase that cleaves glycosidic bonds, reversing the action of PARP enzymes and returning proteins to their native state [1–9].

The precise biological functions of PARG involve the pADPr cycling required for structural chromatin remodeling during DNA repair, transcription, DNA replication and various cell death pathways, ranging from necrosis to apoptosis and autophagy [1,7,10]. In addition, some authors have suggested that the concerted action of PARG and ADP-ribose pyrophosphorylase is capable of generating ATP from ADP-ribose units [11].

*Trypanosoma cruzi*, the etiologic agent of Chagas’ disease, is transmitted by insect vectors when these blood-sucking triatomines deposit on the animal or human skin their feces, containing the infective form of the parasite. Establishment of infection by *T. cruzi* depends on a series of events where cell invasion is a crucial step. Great progress has been made towards understanding the mammalian cell invasion by this pathogen, but still a great deal of work needs to be done in order to draw a complete picture of this complex process.

We have previously characterized PARP from *Trypanosoma cruzi* (TcPARP) and, as opposed to humans and other organisms, both *T. cruzi* and *Trypanosoma brucei* have only one PARP [12]. Exposure of *T. cruzi* epimastigotes to DNA-damaging agents shows a drastic increase in the levels of pADPr in the nucleus, thus confirming pADPr synthesis *in vivo* and suggesting a physiological role for PARP in the trypanosomatid DNA repair signaling process [13]. We have also demonstrated that inhibition of PARP reduces epimastigote growth in culture and affects cell infection by *T. cruzi* [14]. *Trypanosoma cruzi* PARG (TcPARG) has been identified in our laboratory using a database search strategy in a way similar to that described for trypanosomatid PARPs. Here we demonstrate that inhibition of TcPARG causes a delay in cell cycle progression and what is more significant, PARG from the host cell has shown to be essential for the vital cycle of this parasite, pointing out this process requires pADPr degradation and therefore, can be considered as a plausible approach for hindering the infection.

## Materials and Methods

### Materials

All restriction endonucleases and DNA Polymerase Taq were from New England Biolabs Inc., Beverly, MA. Bacto-tryptose and liver infusion were from Difco Laboratories, Detroit, MI. All other reagents were purchased from Sigma Chemical Co., St. Louis, MO.

### Parasites cultures and cell extracts

*Trypanosoma cruzi* epimastigote forms (CL Brener) were cultured at 28 °C for 7 days in liver infusion tryptose (LIT) medium (5 g/l liver infusion, 5 g/l bacto-tryptose, 68 mM NaCl, 5.3 mM KCl, 22 mM Na_2_HPO_4_, 0.2% (W/V) glucose, and 0.002% (W/V) hemin) supplemented with 10% (V/V) FCS, 100 U/ml penicillin and 100 mg/l streptomycin. Cell viability was assessed by direct microscopic examination. Cells were harvested by centrifugation at 850xg and 4 °C, washed three times with PBS and resuspended in buffer A: 50 mM Tris–HCl, pH 8.0, 1.0 mM EDTA, 10% (V/V) glycerol, 10 mM 2-mercaptoethanol, containing protease inhibitors: 1 µg/ml trans-epoxysuccinyl-L-leucylamido(4-guanidino) butane (E-64), 1 mM pepstatin A, 1 mM phenylmethylsulfonyl fluoride (PMSF), and 0.1 mM Na-pTosyl-L-lysine chloro-methyl ketone (TLCK). Cells were lyzed in an Ultrasonic Processor Model W385 Sonicator (Heat Systems-Ultrasonic Inc, Plainview, IL, NY, USA) and the whole extract obtained was used as a protein source for Western blot or Dot blot analysis.

### Preparation of DNA and RNA from *T. cruzi*

DNA from *T. cruzi* epimastigotes form was prepared following the protocol previously described by Pereira et al. [15], its integrity assessed by TAE-agarose gel electrophoresis and quantified by spectrophotometry using NanoDrop 1000 (Thermo, Fisher Scientific, Waltham, MA, USA). Total RNA was obtained using the Total RNA isolation (TRIzol) reagent (Invitrogen) according to the manufacturer’s instructions. RNA integrity was assessed by MOPS Formaldehyde Agarose gel electrophoresis and quantified by spectrophotometry using NanoDrop.

### Southern and Northern Blot analysis

Southern blot analysis was performed using 5 µg of DNA previously digested with restriction endonucleases. After the DNA was electrophorezed in a 1% agarose gel, it was transferred to a Hybond N^+^ Nylon membrane (Amersham Pharmacia Biotech, Piscataway, USA) and hybridized at 65 °C in Church’s buffer [1% (W/V) BSA, 7% (W/V) SDS, 1 mM EDTA pH 8, 0.5% (W/V) Na_2_HPO_4_ with a specific probe corresponding to the whole TcPARG gene, obtained by PCR. The analysis by Northern Blot was carried on a 1% formaldehyde-agarose gel on 10 µg of total RNA. After electrophoresis, it was transferred and hybridized as described in the Southern blot analysis. Blots were subjected to sequential stringent washes at 65 °C and either exposed to AGFA CPBU NEW films (AGFA Gevaert N.V., Belgium) or scanned using a phosphoimager STORM 820 (Amersham, Pharmacia, USA). All probes were labeled with [^32^P] dCTP using Random Primer Extension Labeling System (PerkinElmer LAS, Inc., Boston, MA, USA), according to the manufacturer’s instructions.

### Culture synchronization and cell cycle analysis

*Trypanosoma cruzi* epimastigote cultures were synchronized as previously described by Galanti and co-workers [16]. Briefly, cultures with a density of 10^7^ parasites/ml were incubated in the presence of hydroxyurea (HU) 15 mM for 20 hs. After the incubation period, parasites were washed 3 times with PBS and resuspended in 1 volume of fresh LIT medium. Samples were drawn periodically and analyzed by flow cytometry.

The method used for DNA labeling for flow cytometry analysis was based on the use of propidium iodide (PI, Sigma Chemical Co., St. Louis, MO) staining. The PI fluorescence of individual cell nuclei was measured using a BD FACSAriaTM flow cytometer (Becton Dickinson, San Jose, CA). At least 10^4^ cells of each sample were analyzed. All measurements were made using the same instrument settings.

### Subcellular localization

#### Immunofluorescence

Anti-TcPARG antibodies were obtained as previously described [13]. Parasites were fixed with 3.8% (W/V) formaldehyde in PBS at 4^°^C, permeabilized with fresh PBS-0.1%Triton X-100 and blocked for 1 h at room temperature. PARG was detected with 1:500 primary antibody followed by 1:600 Alexa Fluor 488 goat anti-mouse IgG antibody (Invitrogen). Excess of antibody was removed by 3×5-min washes in PBS, and the nuclei were stained with 2 µg/ml DAPI (Sigma) in PBS. Coverslips were washed with distilled water and mounted in Mowiol and then visualized using an Olympus BX41 microscope.

#### Electron microscopy

About 10^8^ epimastigote cells were harvested and washed twice in PBS. The parasites were fixed in PBS 2.5% glutaraldehyde, 4% formaldehyde, pH 7.3 for 1 h and then embedded in epoxy resin, sectioned, and stained using 1:50 mouse polyclonal TcPARG antibody followed by 1:100 anti-mouse antibody conjugated with 10-nm gold particle (GE healthcare, Little Chalfont, Buckinghamshire, UK). Images were obtained on a Zeiss EM 10 C transmission electron microscope operating at 80 kV.

### Western blot and Dot Blot analysis

For Western blot analysis, the protein in the whole cell lysate was quantified by Bradford and 35 µg of protein were electrophorezed on 10% SDS-PAGE gel and transferred to Amersham Hybond-ECL nitrocellulose membrane (GE healthcare, Little Chalfont, Buckinghamshire, UK), according to the manufacturer’s instructions. TcPARG was detected with an anti-TcPARG (1:10000) specific antiserum, followed by anti-mouse horseradish peroxidase-conjugated antibody (1:6000) (Kirkegaard Perry Laboratories, Inc.). β-tubulin was used as loading control. For Dot blot analysis, 5 µg of protein was spotted onto an Amersham Hybond-ECL nitrocellulose membrane (GE healthcare) by using a Minifold Dot Blot System (Schleicher Schuell, Inc.). Immunodetection of ADP-ribose polymers was carried out using mouse polyclonal antibody directed against the pADPr (1:5000) (BD), followed by anti-rabbit horseradish peroxidase-conjugated antibody (1:6000) (Kirkegaard Perry Laboratories, Inc.).

The signal was detected with the Western Lightning Plus-ECL kit (PerkinElmer).

### In vivo inhibition of *Trypanosoma cruzi* PARG

For the assessment of the effect of DEA (6,9-diamino-2-ethoxyacridine lactate monohydrate) (Trevigen, Inc.) on PARG activity, *T. cruzi* epimastigotes were grown in LIT complete medium for 4 days up to a parasite density of 10^7^ parasites/ml in the absence or presence of DEA. Cells were harvested by centrifugation at 1500×g, washed with PBS and the whole extract, obtained as described above, was used for Dot blot assay.

In the case of ADP-HPD (adenosine diphosphate (hydroxymethyl) pyrrolidinediol) inhibitor (Calbiochem), *T. cruzi* epimastigotes were grown in LIT complete medium for 4 days up to a parasite density of 10^7^ parasites/ml, collected by centrifugation at 750×g for 5 minutes and resuspended in PBS-Glucose 2%. Parasites were pre-incubated in the presence of the PARG inhibitor, ADP-HPD, for 30 minutes and treated with 300 µM hydrogen peroxide for 10 minutes. Cells were washed to remove the oxidizing agent. After 30 and 60 minutes post-agent removal, parasites were harvested and lysed and the whole extract used for Dot blot assay as described above.

### Growth inhibition assays

*T. cruzi* epimastigotes were grown in LIT complete medium for 4 days up to a parasite density of 10^7^ parasites/ml. The culture was then placed in 96-well sterile plates in 100 µl aliquots and PARG inhibitors (DEA or ADP-HPD) were added to previous digitonin-permeabilized-cells. DMSO was used as a control at 1% V/V concentration. The number of epimastigotes was determined daily by counting formaldehyde-fixed parasites in a Neubauer chamber. In all experiments performed, conditions were tested in triplicates. Day 0 corresponds to the day in which the cultures were placed in the plate and the inhibitors added. Significance of the results was analyzed with one-way ANOVA using GraphPad Prism version 5.03 for Windows (GraphPad Software).

#### *Trypanosoma cruzi* infection of Vero and A549 cells

Vero cells were cultured in D-MEM medium (Gibco), supplemented with 2 mM L-glutamine, 10% (V/V) FCS, 100 U/ml penicillin and 100 mg/l streptomycin. Wild type and PARG silenced (shPARG) pulmonary adenocarcinoma A549 cell lines were provided by Dr. Virág and Dr. Erdélyi, from the University of Debrecen, Hungary [8]. A549 cells were grown in RPMI-1640 medium (Gibco), supplemented with 2 mM L-glutamine, 1 mM Sodium Pyruvate, 10% (V/V) FCS, 100 U/ml penicillin and 100 mg/l streptomycin.

Trypomastigotes were collected by centrifugation of the supernatant of previously infected cultures at 1500×g at room temperature for 7 minutes and incubated for 3 hours at 37 °C in order to allow the trypomastigotes to move from the pellet into the supernatant. After this period, the supernatant was collected and trypomastigotes were counted in a Neubauer chamber. The purified trypomastigotes were pre-incubated in the presence or absence of 1 µM DEA for 30 minutes and then used to infect new monolayers of Vero or A549 cells. For this, 50 trypomastigotes/cell were added to the medium of 24 hour-old monolayers and incubated for 24 hours at 37 °C, after which they were removed by changing the cell culture medium.

The infection was allowed to proceed and growth medium was changed periodically during the first 5 days. In the PARG inhibited samples, DEA was kept in the growth medium at 1 µM throughout the experiment. At the indicated days, cells were fixed and stained by May Grünwald Giemsa technique. Cells were visualized using an Olympus BX41 microscope. Amastigotes and host cells were counted using the ImageJ software in at least 7 microscopic fields. Alternatively, trypomastigotes in the supernatant of the cell cultures were counted without prior fixation on a Neubauer Chamber at the indicated days. All experiments were performed in triplicates. Significance of the results was analyzed with two-way ANOVA using GraphPad Prism version 5.03 for Windows (GraphPad Software).

For the experiments in which only trypomastigotes were subjected to the effect of DEA, this infective form was obtained as above described and incubated in the presence of DEA for 4 hours, then collected by centrifugation and resuspended in fresh medium without DEA. These trypomastigotes were used to infect wild type Vero cells in the absence of DEA, following the infection protocol recently addressed. At 72 and 96 hours post-infection, cells were fixed and stained by May Grünwald Giemsa technique.

## Results

### *In silico* analysis of TcPARG

**Figure 1:**
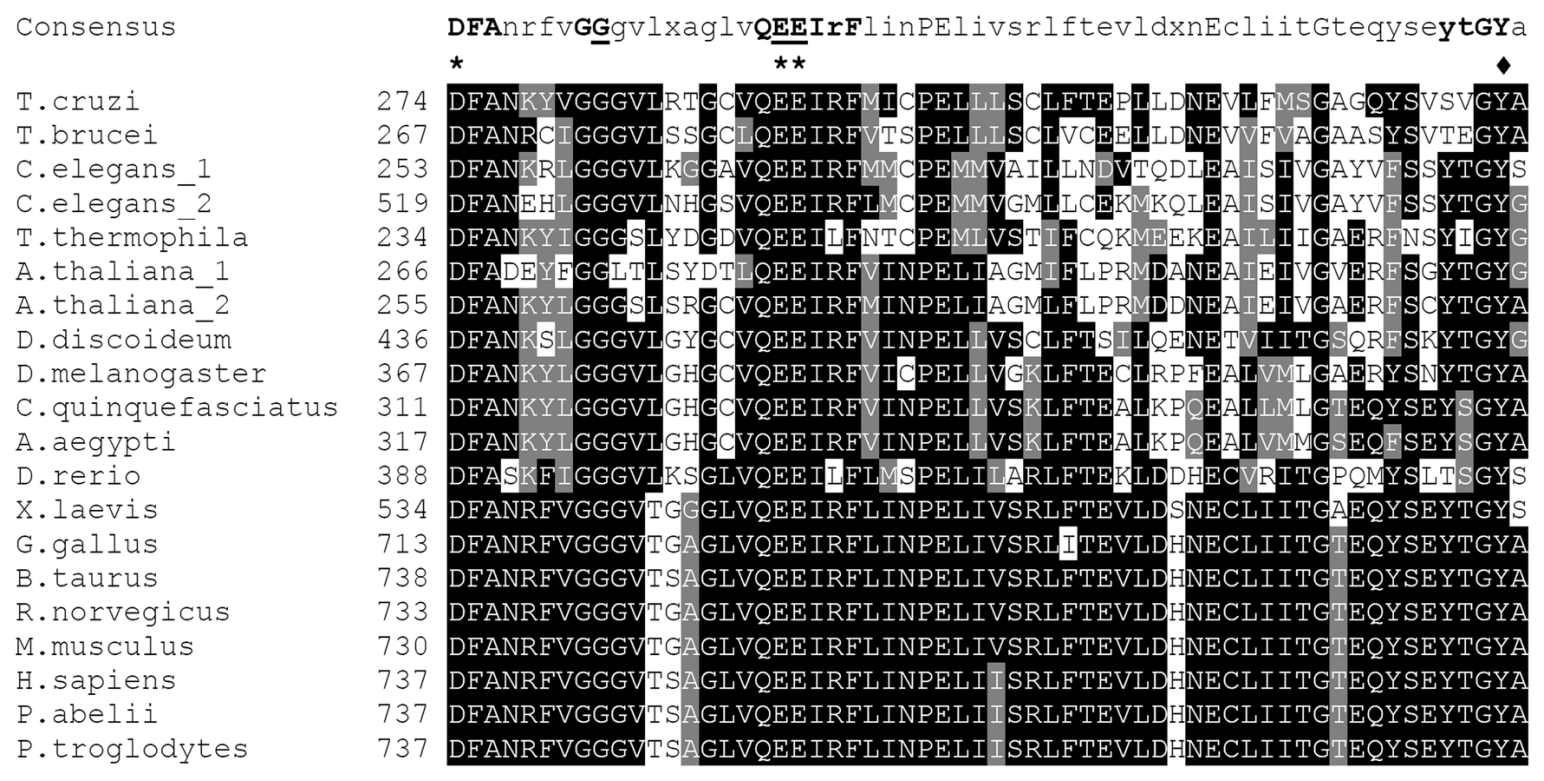
Amino acid sequence alignment of the PARG signature from different organisms. The multiple alignment of the PARG signature amino acid sequences corresponding to *T. cruzi* PARG (accession number ABG73229); *T. brucei* PARG (GeneDB Systematic Name: Tb09.211.3760); *C. elegans*_1 PARG (accession number NP_501496) and *C. elegans*_2 PARG (accession number NP_501508); *T* *. thermophila* (accession number EAR94344); *A. thaliana*_1 PARG (accession number NP_973578); *A. thaliana*_2 PARG (accession number AAK72256); *D. discoideum* PARG (accession number XP_642024); *D. melanogaster* PARG (accession number NP_477321); *C* *. quinquefasciatus* PARG (accession number XP_001853435); *A. aegypti* PARG (accession number XP_001659301); *D. rerio* PARG (accession number XP_001338257); *X. laevis* PARG (accession number NP_001089602); *G. gallus* PARG (accession number XP_421502); *B. taurus* PARG (accession number NP_776563); *R. norvegicus* PARG (accession number NP_112629); *M. musculus* PARG (accession number NP_036090); *H. sapiens* PARG (accession number NP_003622); *P. abel*ii PARG (accession number NP_001125086); *P* *. troglodytes* PARG (accession number XP_001139727) was generated with the ClustalW2 program and edited with the BOXSHADE (3.21) software. Colors used for amino acids background are as follow: white for different residues, black for identical residues, gray for similar and conserved residues. Asterisk: essential acidic residues D-E-E, underlined: key residues, G and two consecutive E, and black diamond: important Y residue.

*T. cruzi* PARG was identified in our laboratory using a database search strategy in a way similar to that we have previously described for trypanosomatid PARPs [12]. The search carried on the *T. cruzi* databases showed one possible ORF coding for a putative PARG and the corresponding sequence was annotated in GenBank under Accession Number DQ679799. A similar search performed on the *Trypanosoma brucei* genomic database showed a putative sequence (Tb09.211.3760), but in the related trypanosomatid *Leishmania major* no positive hits were identified, outcome that agrees with the lack of a gene coding for poly(ADP-ribose) polymerase. Based on the information obtained from the databases, TcPARG codes for a protein of 540 amino acids. We compared *T. cruzi* and *T. brucei* glycohydrolase amino acid sequences by using alignments that cover the full length of the sequences (global alignments), and found 46.5% of identity and 60% of similarity. In addition, sequence alignment of the PARG catalytic domain fragment, known as PARG signature, showed a higher conservation of this motif in trypanosomatid PARGs (61.7% and 68.3% of identity when comparing TcPARG with *Homo sapiens* PARG and the orthologous sequence in *Drosophila melanogaster*, respectively). The three essential acidic residues (D-E-E) identified within the PARG catalytic fragment [17] are present in both TcPARG and TbPARG (Figure 1, asterisks). These two consecutive glutamates and a glycine (Figure 1, underlined) were also reported as key residues by Slade et al. [18]. In addition, a tyrosine residue (Figure 1, diamond) implicated in the binding to the PARG inhibitor ADP-HPD is also present in TcPARG. When a wide region including the complete PARG catalytic motif was aligned, the identity between *T. cruzi* and *T. brucei* PARG value was 51.5% and about 30% when compared to the other organisms (Table 1).

**Table 1:** Identity/similarity percentage of *T. cruzi* and *T. brucei* PARG catalytic domain.

	***T. cruzi*_PARG**	***T. brucei*_PARG**
***T. cruzi*_PARG**	------	**51.5/65.1**
***T. brucei*_PARG**	**51.5/65.1**	------
***A. thaliana*_*1*_PARG**	**26.8/39.1**	**26.4/38.0**
***A. thaliana*_*2*_PARG**	**27.9/39.5**	**27.5/38.8**
***C. elegans*_*1*_PARG**	**24.4/38.6**	**25.7/41.3**
***C. elegans*_*2*_PARG**	**28.2/40.3**	**26.4/39.8**
***B. taurus*_PARG**	**32.6/47.7**	**33.9/47.3**
***H. sapiens*_PARG**	**32.9/48.8**	**34.2/47.8**
***D. melanogaster*_PARG**	**33.9/46.0**	**33.7/46.6**
***R. norvegicus*_PARG**	**32.9/48.8**	**33.7/47.3**
***M. musculus*_PARG**	**32.9/48.8**	**33.9/47.6**
***G. gallus*_PARG**	**32.3/46.5**	**35.0/47.9**
** *C* *. quinquefasciatus* _PARG**	**34.3/46.9**	**34.5/46.2**
***D. discoideum*_PARG**	**33.6/49.4**	**30.9/46.2**
***A. aegypti*_PARG**	**34.0/44.2**	**34.3/46.9**
***D. rerio*_PARG**	**29.4/47.2**	**30.1/46.1**
** *P* *. abelii* _PARG**	**32.9/48.8**	**34.2/47.8**
***X. laevis*_PARG**	**32.0/45.2**	**35.0/47.7**
** *T* *. thermophila* _PARG**	**28.7/45.9**	**26.4/43.6**
** *P* *. troglodytes* _PARG**	**32.7/48.5**	**33.2/46.2**

*T. cruzi* and *T. brucei* PARG catalytic motif amino acid sequences were aligned with the catalytic regions of PARGs from different organisms. The optimum alignments were generated using the EMBOSS: needle online version at the European Bioinformatics Institute which applies the Needleman-Wunsch global alignment algorithm. *T. cruzi* PARG (accession number ABG73229); *T. brucei* PARG (GeneDB Systematic Name: Tb09.211.3760); *C. elegans*_1 PARG (accession number NP_501496) and *C. elegans*_2 PARG (accession number NP_501508); *T* *. thermophila* (accession number EAR94344); *A. thaliana*_1 PARG (accession number NP_973578); *A. thaliana*_2 PARG (accession number AAK72256); *D. discoideum* PARG (accession number XP_642024); *D. melanogaster* PARG (accession number NP_477321); *C* *. quinquefasciatus* PARG (accession number XP_001853435); *A. aegypti* PARG (accession number XP_001659301); *D. rerio* PARG (accession number XP_001338257); *X. laevis* PARG (accession number NP_001089602); *G. gallus* PARG (accession number XP_421502); *B. taurus* PARG (accession number NP_776563); *R. norvegicus* PARG (accession number NP_112629); *M. musculus* PARG (accession number NP_036090); *H. sapiens* PARG (accession number NP_003622); *P* *. abelii* PARG (accession number NP_001125086); *P* *. troglodytes* PARG (accession number XP_001139727).

### Genetic organization and expression of PARG in *Trypanosoma cruzi*

**Figure 2:**
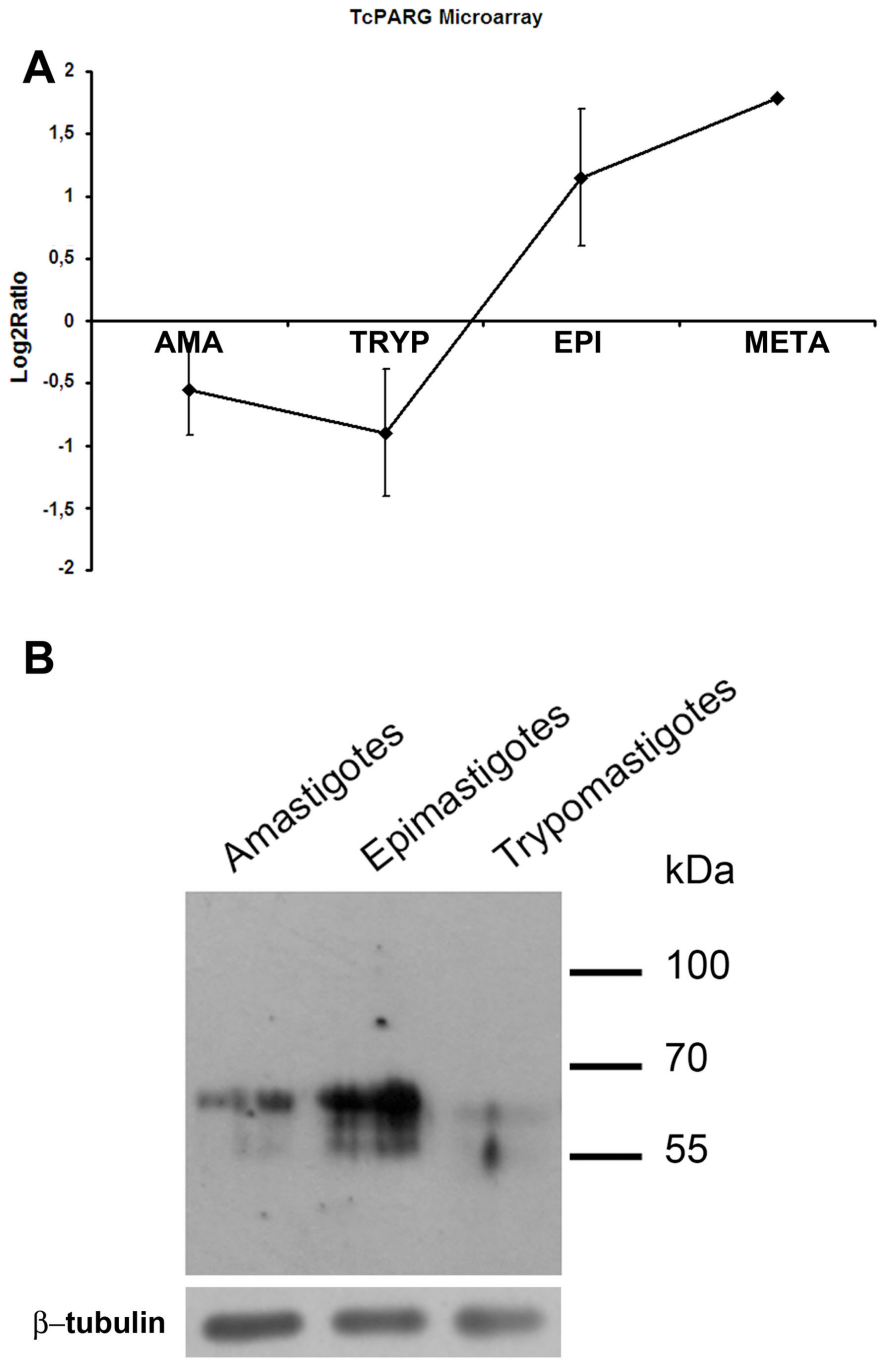
Expression of TcPARG throughout the *Trypanosoma cruzi* life-cycle. (A) Microarray expression data for TcPARG over the course of *T. cruzi* life-cycle. TcPARG mRNA relative abundance was evaluated by using the transcriptome analysis of different *T. cruzi* stages available at Gene Expression Omnibus database (www.ncbi.nlm.nih.gov/geo, DataSets: GSE14641). Shown are mean microarray log_2_ ratios (stage/reference) for TS significantly regulated in *Trypanosoma cruzi* amastigotes (AMA), trypomastigotes (TRYP), epimastigotes (EPI), and metacyclic trypomastigotes (META). (B) Western blot analysis of the three life-cycle stages of *T. cruzi*. Protein extracts (35 µg) of amastigote, epimastigote or trypomastigote stages of *T. cruzi* were solved in a 10% polyacrylamide gel, transfer to a nitrocellulose membrane and revealed with an anti-TcPARG (1:10000) specific antiserum. β-tubulin was used as loading control.

The copy number of PARG genes was determined by Southern blot; results show that TcPARG is a single-copy gene (data not shown), which is consistent with the results obtained by searching at the GeneDB database (http://www.genedb.org). We found the presence of only one transcript by Northern blot analysis on epimastigote mRNA obtained at different time points throughout the cell cycle in synchronized cultures, using the full length sequence as a probe (Figure S1 A). The analysis of the expression of the TcPARG protein on synchronized epimastigotes also showed its presence in every cell cycle phase, as evidenced by Western blot using specific anti-TcPARG antisera. It can be seen that an increase in PARG protein levels occurs up to eight hours after hydroxyurea relief. Later, lower molecular mass bands were also detected (Figure S1 B). TcPARG mRNA relative abundance over the course of *T. cruzi* life-cycle was evaluated as well, by using the Gene Expression Omnibus database (www.ncbi.nlm.nih.gov/geo, DataSets: GSE14641) generated by Minning and co-workers [19]. This study showed that in mammalian-host stages (amastigotes and trypomastigotes) TcPARG mRNA is down-regulated, while in the insects stage form (epimastigotes) it is up-regulated (Figure 2 A). The results of these analyses were compared to the protein expression pattern determined by Western blot in these three life-cycle stages of *T. cruzi*. In accordance, we found a strong correlation between the specific transcript abundance and protein relative levels (Figure 2 B). Note that the expression of TcPARG shows consistency between the stages of the same host, despite the fact that previous genomic studies of kinetoplastids have found a low proportion of their genomes to be stage regulated.

### TcPARG is targeted to the nucleus

**Figure 3:**
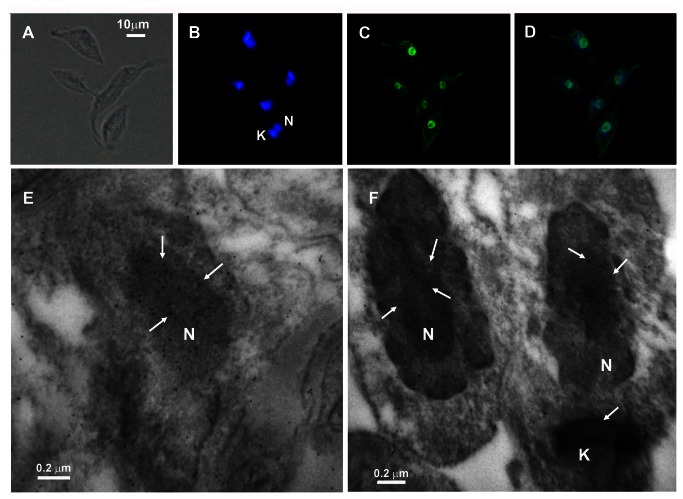
Immunolocalization of PARG on *Trypanosoma cruzi*, CL Brener epimastigotes. The parasites were fixed for 25 min with 3.8% (W/V) formaldehyde in PBS at 4°C, permeabilized with fresh PBS - 0,1% Triton X-100 and blocked for 1 h at room temperature with 5% (W/V) BSA in PBS. (A) Differential interference contrast (DIC). (B) Cells were counterstained with DAPI to identify nuclear DNA and kinetoplastid DNA. (C) PARG was detected with 1:500 mouse polyclonal TcPARG antibody followed by 1:600 Alexa Fluor 488 goat anti-mouse IgG antibody. (D) Merge of PARG and DNA signals show the nuclear localization of this enzyme. Bar: 10 µm. (E–F) For electron microscopy, epimastigotes were fixed in PBS 2.5% glutaraldehyde, 4% formaldehyde, embedded in epoxy resin and PARG detected with 1:50 mouse polyclonal TcPARG antibody followed by 1:100 anti-mouse antibody conjugated with 10-nm gold particle. N: nucleus; K: kinetoplast. Bar: 0.2 µm.

Using previously obtained specific antibodies [13], we assessed the localization of the enzyme in the parasite by immunofluorescence and electron microscopy. TcPARG was present in the nucleus both in the absence (Figure 3 A–D) or presence of DNA damage (data not shown). These results were corroborated on a transgenic *T. cruzi* line expressing a copy of TcPARG which had been fused to the RED protein (Figure S2). Similar results were obtained when we attempted to ascertain the localization of TcPARG in different cell cycle stages using the antibody directed against TcPARG on wild type epimastigotes coming from synchronized cultures (Figure S1 C) or in the intracellular amastigote (data not shown). TcPARG showed to be localized in the nuclear compartment in every condition tested. We also examined the localization of this enzyme by transmission electron microscopy. Results in Figure 3 E-F show that TcPARG is localized to the nucleus as can be seen by the high density of associated immunogold particles (arrows). We were not able to detect this enzyme in other subcellular compartment although a slight immunogold signal was visualized associated to the kinetoplast.

### In vivo inhibition of *Trypanosoma cruzi* PARG

**Figure 4:**
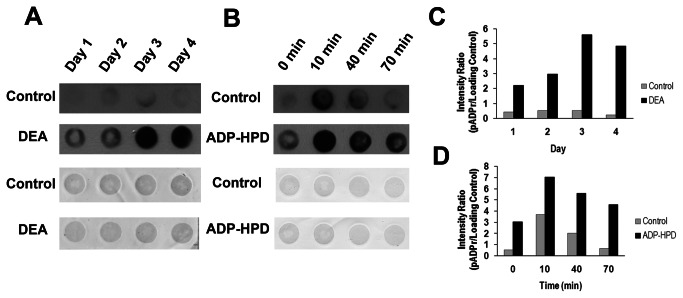
Inhibition of PARG activity in *Trypanosoma cruzi*. (A) Dot Blot analysis of poly(ADP-ribose) accumulation during epimastigote growth in cultures untreated (Control) or treated with 1 µM of the PARG inhibitor DEA (DEA) for four days. (B) Dot Blot analysis of poly(ADP-ribose) formation and degradation after a genotoxic stimulus in untreated epimastigotes (Control) or in parasites preincubated with 1 µM of the PARG inhibitor ADP-HPD (ADP-HPD) for 30 min. After preincubation, hydrogen peroxide 300 µM was added (0 min), incubated for 10 minutes at 28^°^C (10 min) and the oxidizing agent was removed. Samples were obtained at 30 and 60 minutes post agent removal (indicated as 40 min and 70 min respectively). Five µg of total protein extract were dotted onto nitrocellulose membrane and an anti-pADPr polyclonal antibody was used to detect the formed polymers (upper panels). Ponceau Red was used to detect the whole protein sample in each dot (lower panels). (C–D) Data were normalized to protein content and are shown as the ratio of pADPr to loading control signals. Representative experiment of three independent assays.

We measured the presence of ADP-ribose polymer in epimastigotes in culture until the 4th day of growth. As shown in Figure 4 A, a slight amount of pADPr was observed in control conditions as a result of physiological pADPr metabolism during growth. In the presence of DEA, a known PARG inhibitor, an enhanced polymer accumulation was observed, probably due to a lower rate in the catabolism of this molecule (Figure 4 A and C). ADP-HPD, an analogue of the ADP-ribose product of the PARG-catalyzed reaction, is one of the most potent and widely used PARG inhibitor, despite its lack of cell permeability. In order to evaluate the affectivity of this more specific PARG inhibitor in these organisms, we measured pADPr formation after genotoxic insult in *T. cruzi* epimastigotes previously permeabilized and incubated with this compound. Figure 4 B shows pADPr formation after epimastigote treatment with H_2_O_2_. It can be seen that the presence of the inhibitor leads to a higher accumulation of the polymer after exposure to a genotoxic stimulus (Figure 4 B and D).

### Role of PARG in *T. cruzi* proliferation

**Figure 5:**
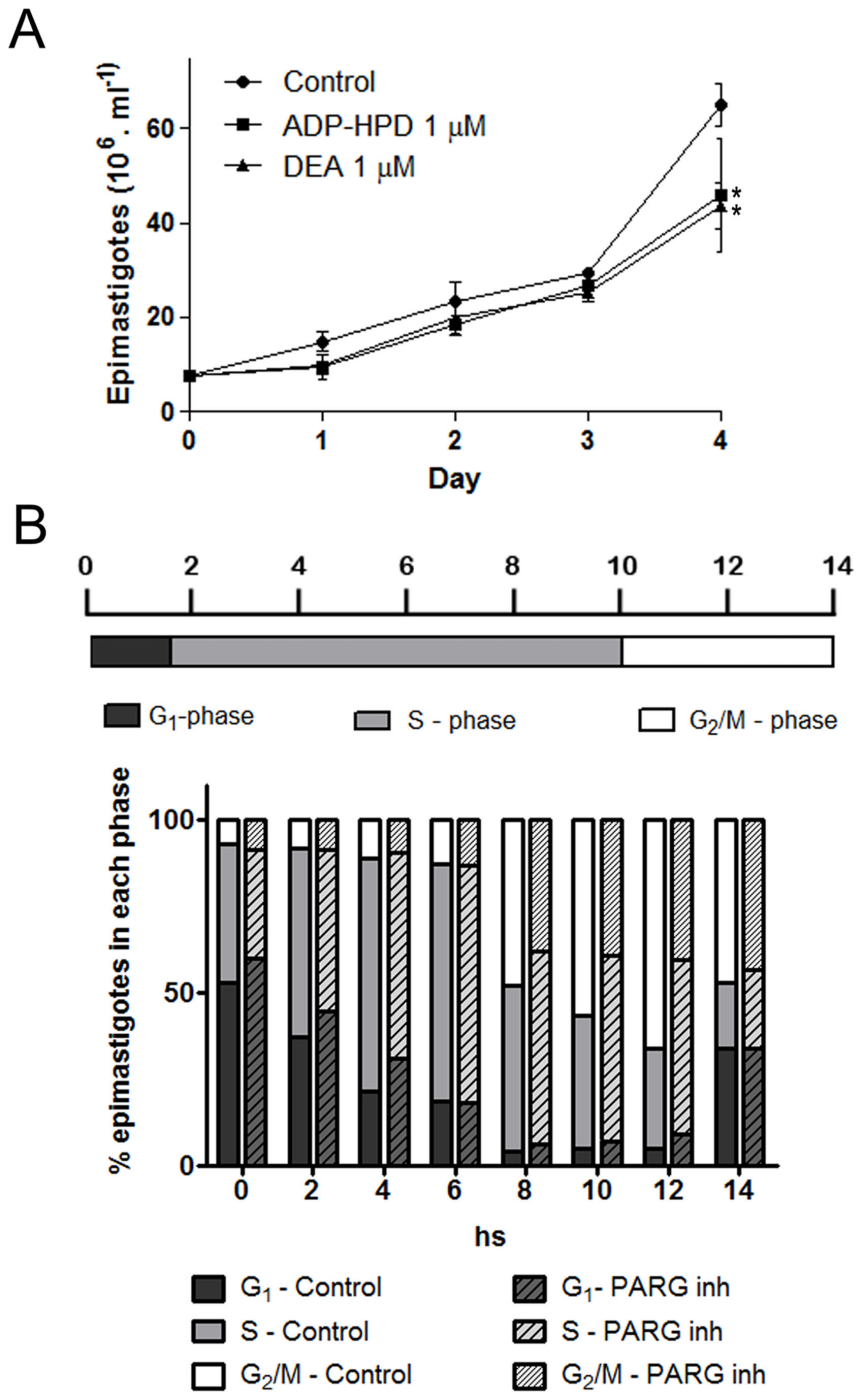
Role of TcPARG in *Trypanosoma cruzi* epimastigotes proliferation and cell cycle progression. A) Effect of the PARG inhibitors ADP-HPD and DEA on *T. cruzi* growth and survival was determined by incubating epimastigotes at an initial density of 10^7^ parasites/ml in the continuous presence of inhibitors at 1 µM. The number of epimastigotes was determined daily by counting formaldehyde-fixed parasites in a Neubauer chamber. All data points were determined in triplicates and shown as means with standard deviation. The significance of the results versus the control at day 4 was analyzed with t test and indicated in the figure (* p0.05). B) Effect of ADP-HPD at 1 µM concentration on cell cycle progression of epimastigotes was determined by adding the inhibitor at the indicated concentration to the culture media of hydroxyurea synchronized parasites after digitonin permeabilization. Samples were drawn every 2 hours for 14 hours and DNA content was determined by propidium iodide staining followed by flow cytometry analysis. The percentage of epimastigotes in each cell cycle phase was determined by setting gates according to the DNA content in the 0 hs of the control sample and maintained for all other samples. The data were analyzed using the Cyflogic software.

To further characterize the importance of PARG in *T. cruzi* survival, we evaluated the effect of PARG inhibitors on epimastigote cell growth in culture. All the inhibitors assayed at 1 µM concentration were able to diminish the growth rate in the conditions here tested, the reduction being 35.7 and 37.5% at day 4 of growth for ADP-HPD and DEA respectively, as compared to control conditions without the addition of inhibitor (Figure 5 A).

When the effect of ADP-HPD was evaluated on cell cycle progression by flow cytometry, cells incubated in the presence of PARG inhibitor presented differences in the percentage of epimastigotes in the S and G2/M-phases at 8, 10 and 12 hours after the culture was released from the HU-imposed arrest. While control cultures showed a constant increase in the fraction of epimastigotes in the G2/M-phase in the aforementioned period, the number of cells in this cell cycle phase, as well as in the S-phase, in PARG-inhibited cultures remained unaltered throughout this period (Figure 5 B). Nevertheless, both cultures could continue with cell cycle progression and entry into a new round of replication.

### Inhibition of PARG interferes with cell invasion and the progress of infection

**Figure 6:**
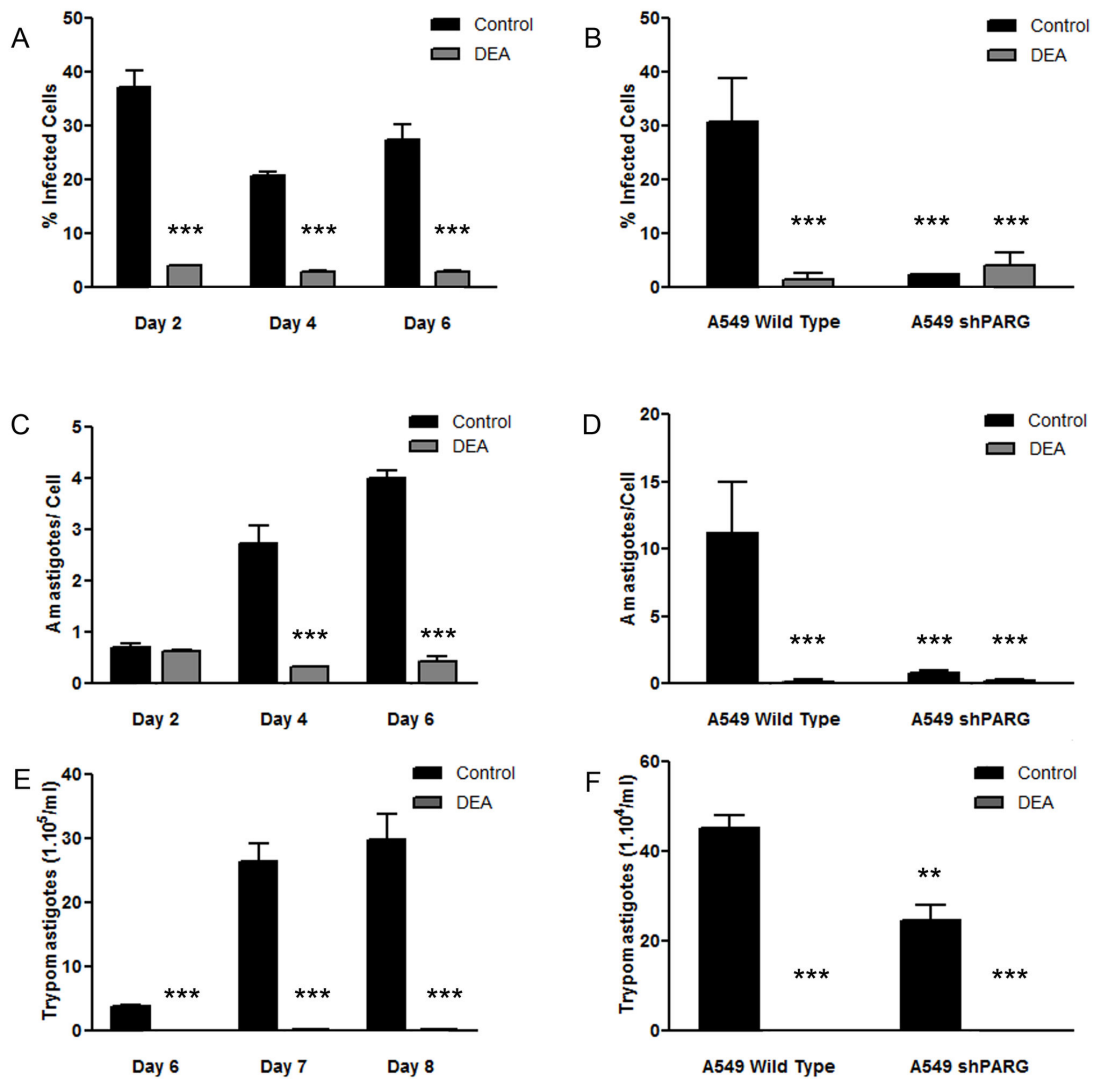
Effect of PARG inhibitors on *T. cruzi* infection on Vero or A549 host cells. *T. cruzi* trypomastigotes were purified from the supernatant of previously infected cells and preincubated for 30 min in the respective culture medium in the absence (Control) or presence of 1 µM PARG inhibitor (DEA). Twenty-four hours Vero, A549 wild type or shPARG (hPARG silenced) cell monolayers were infected with 50 trypomastigotes/cell. The infection process was followed by microscopic direct visualization. At the indicated days (A and C) or at day 6 post-infection (B and D), percentage of infected cells and number of amastigotes intracellular were determined on May-Grünwald Giemsa stained samples. Amastigotes and cells were counted using the ImageJ software in at least 7 fields. The number of trypomastigotes/ml in the supernatant of infected cell cultures was determined by counting unfixed trypomastigotes in a Neubauer chamber at the indicated days (E) or at day 9 post-infection (F). All points were determined in triplicates and shown as means with standard deviation. Significance of the result versus the Control (***p0.001; two way ANOVA) or Wild Type Control (***p0.001; **, p0.01; two way ANOVA) is indicated.

The inhibitor DEA was used in *in vitro* cell infection experiments, using Vero cell line as host cells. Controls, in which uninfected cells were incubated in the presence or absence of DEA, showed that this compound did not alter the growth of the host cell line (data not shown). The results in Figure 6 A show that the percentage of infected cells in control conditions was about 40% at day two post infection. The level of infection diminished at day four probably due to a differential cell division rate and then increased again as the result of the reinfection process perpetrated by the trypomastigotes released. This observation was supported by the presence of trypomastigotes in the supernatant at day 6 (Figure 6 E). When DEA was present in the culture medium, not only the percentage of infected cells was reduced but also the amount of amastigotes per cell was greatly diminished, avoiding the establishment of the infection (Figure 6 A and C and Figure S3). These results suggest that both invasion and proliferation of intracellular stage of the parasite could be interrupted in the presence of the PARG inhibitor.

Since DEA could potentially be inhibiting either the parasite enzyme or the host cell enzyme, we performed a similar experiment using the human cell line A549 with stably suppressed PARG (A549 shPARG) as host cell. Knock down of PARG had no effect on normal cell growth as was stated by Erderlyi et al. [8] A549 shPARG showed a drastically reduction in both the percentage of infected cells and in the number of amastigotes per cell (Figure 6 B and D). The results obtained in PARG silenced cells showed that the presence of this enzyme is necessary in the host cell for the progression of the infection.

As a complementary analysis we evaluated the effect of TcPARG inhibition by pre-incubation of trypomastigotes in the presence of DEA for four hours previous to the initial infection, but the PARG inhibitor was not added to the cell culture medium afterwards. The percentage of wild type Vero infected cells showed no significant differences when compared to control infection conditions (no pre-incubation of trypomastigotes or addition of DEA to the culture medium) at 72 and 96 hs post-infection (data not shown).

It is worth mentioning that the wild type A549 cell line showed, at day six after infection, a higher number of amastigotes per cell than Vero cell cultures in the absence of DEA (Figure 6 C and D). However, when A549 cells were treated with DEA, the reduction in the number of amastigotes per cells was similar to the results obtained when A549 PARG silenced cells were infected, showing no further effect over those already produced by the absence of this enzyme in the host cell (Figure 6 D and Figure S4). Finally, there was a clear decrease in the trypomastigote number in all DEA treated cells and in the silenced PARG cell line, when compared to their respective controls (Figure 6 E-F). Interestingly, when the presence of trypomastigotes in the supernatant culture media of shPARG A549 cells was evaluated, it showed that DEA treatment significantly reduced the trypomastigote concentration (Figure 6 F). Taking into account that the number of trypomastigotes in the supernatant was the only affected parameter when PARG silenced cells were treated with DEA but that pre-incubation of trypomastigotes in this PARG inhibitor has no effect on the initial infection effectivity of these parasites on Vero cells, we hypothesized that DEA could have a direct effect over *T. cruzi* amastigote intracellular replication or amastigote to trypomastigote differentiation processes, as well as over the host cell.

## Discussion

We have previously reported the presence of a poly(ADP-ribose) polymerase in the trypanosomatid *Trypanosoma cruzi*. Moreover we demonstrated that these parasites posses only one PARP responsible for the synthesis of these polymers and that poly(ADP-ribose) metabolism is involved in the cellular response to the existence of DNA damage in these organisms [12,13]. In contrast to the large body of information related to PARP that is nowadays available, there is less reported data on PARP’s counterpart, poly(ADP-ribose) glycohydrolase. In the present work, we report the PARG expression profile during the life cycle of a kinetoplastid parasite, and relate its activity to cell proliferation.

Recently, Slade et al. [18] described the first crystal structure and the catalytic mechanism of the poly(ADP-ribose) glycohydrolase from *Thermomonospora* *curvata* . The catalytic activity of PARG has been proposed to relay on the two glutamic acid residues in the GGG-X6-8-QEE motif (E114 and E115). Although both residues have been described to be essential for activity, only E115 is proposed to be directly involved in the catalysis. Mutation studies in *T* *. curvata* PARG support the notion that E114 would participate in the correct substrate positioning and binding [18]. Our homology searches revealed that TcPARG protein sequence bears the PARG signature (GGG-X6–8-QEE), which includes the key residues: two consecutive glutamates (E292 and E293) and a glycine (G282). In addition, a tyrosine residue (Y332) implicated in binding to the PARG competitive inhibitor ADP-HPD is also present in TcPARG [17,18,20,21].

Poly-ADPr chains degradation in higher eukaryotes is carried out mainly by PARG, although ARH3 (ADP-ribose hydrolase-like) and ADP-ribosyl protein lyase have also been assigned accessory roles in pADPr decomposition [3,22,23]. Searches performed in our laboratory indicated that the evolutionary unrelated ARH3 proteins would be absent in *T. cruzi* genome. We cannot rule out the possible existence of another structurally and sequence-unrelated hydrolase protein that could be involved in pADPr metabolism in these parasites.

TcPARG expression was demonstrated to be differential between developmental stages in *T. cruzi* and throughout the different phases of the cell cycle. TcPARG protein levels raise up to hour 8 after hydroxyurea arrest relief, period included in the S-phase, in which nuclear DNA and kDNA replication is taking place. Remarkably, in this period as well, larger amounts of pADPr were detected in this trypanosomatid (unpublished data). We have observed, between hours 8-14 in synchronized epimastigotes, the appearance of proteins of lower molecular weights than TcPARG. Given that alternative splicing in these parasites does not occur, this result could be due to specific protease cleavage events as a possible control or to unspecific protein degradation. Affar and co-workers have reported PARG degradation mediated by caspase 3 [24]. Since up to date these proteases were not described in trypanosomatids, we cannot rule out other regulatory mechanisms, which will need to be addressed in the future.

In higher eukaryotes, PARG has been reported to change its subcellular localization in response to DNA damaging agents: in the absence of single strand breaks on the DNA, PARG is found in the cytoplasm of the cell. However, when the cells are exposed to an agent capable of causing lesions on the genetic material, PARG migrates from the cytoplasm into the nucleus, where high amounts of pADPr are produced. This is thought to be a regulatory mechanism of the glycohydrolase activity displayed by this enzyme. As shown in the present work, in *T. cruzi* we have observed the nuclear localization of TcPARG despite the absence of DNA damage. For hPARG, shuttling between nucleus and cytoplasm has been also reported during the cell cycle as well as its localization to the centrosomes in mitosis [25] and different nuclear and extranuclear isoforms have been described [26–28]. Our results are in dissonance with this report since in synchronized epimastigotes we have found that TcPARG was present mainly in the nucleus during all phases, G1, S and G2/M (Figure S1 C). We have previously reported that TcPARP migrates to the nucleus after a genotoxic insult in correlation with the detection of polymer formation in this organelle [13]. The localization of these enzymes could be a strategy for the regulation of the pADPr synthesizing-degradating activity. These results are in agreement with the necessity of PARG for pADPr degradation since pADPr is metabolized mainly by this enzyme. A deeper evaluation of the possible presence of TcPARG in the mitochondrion of *T. cruzi* needs to be addressed. It is possible that this enzyme could be implicated in the regulation of mitochondrial enzymes, since we have obtained many of these proteins pADPrilated by immunoprecipitation, even in the absence of damaging agents (unpublished data).

The signal that leads these enzymes to its particular localization has not been determined, precise subcellular localization motifs have not been described yet in trypanosomatids. This finding posses a new question regarding the mechanism through which the activity of PARG and PARP is regulated in these parasites. Further studies will be necessary to find the signals that control the subcellular localization of both TcPARP and TcPARG, in order to better understand the particular distribution of these enzymes.

The PARG inhibitors tested here had an impact on *T. cruzi* epimastigotes growth ratio, at least in culture. The unspecific inhibitor DEA [29] reduced parasite growth by about 35% at concentrations of 1 µM. Although this compound might be affecting other cellular processes, these results were supported by the similar effect observed when the more specific inhibitor ADP-HPD [30] was used. This effect is observed on epimastigote cultures after 4 days of incubation in the presence of the PARG-inhibiting compounds, pointing out that the reduction of the growth ratio might be related to the accumulation of DNA damages throughout time due to the lack of an appropriately functioning pADPr metabolism. The diminished growth ratio displayed by the epimastigotes incubated in the presence of PARG inhibitors has shown to be independent of the culture phase, since both log-phase and stationary phase cultures showed reduced growth only after the 4-day incubation period.

The impact of DEA and ADP-HPD on epimastigote growth ratio is moderate, showing only a partial reduction of epimastigotes ability to survive or duplicate. It has been pointed out that PARG inhibitors show low cell permeability [31,32] and we have also encountered a partial inhibition of pADPr degradation *in vivo* in the presence of these compounds, what may explain the results here obtained.

As we have shown previously, TcPARP activation also leads to automodification by ADP-ribosylation, which results in PARP inhibition [12]. PARG is involved in the removal of polymers from different proteins, including PARP itself; in this regard, inhibition of PARG could extend the half-life of polymers on TcPARP and other proteins in the nucleus and hence modify cell cycle progression. As reported in this work, the presence of PARG inhibitors caused an alteration in the progression of the cell cycle in *T. cruzi* epimastigotes. This result is in agreement with those obtained for 3-aminobenzamide-treated cells, in which PARP was inhibited and a lower growth rate was observed, suggesting that pADPr metabolism could play a role in the earlier phase of epimastigotes growth, even in the absence of exogenous DNA damage [13]. Therefore, an explanation for the data here obtained is that inhibiting TcPARG leads to the depletion of the pool of non-modified TcPARP available to repair other sites of damage that are spontaneously occurring. Thus, an indirect effect of PARG inhibition is the shutdown of PARP activity. Studies on PARG knockout mice suggest that absence of PARG activity may represent a strategy to enhance the effectiveness of chemotherapeutic agents and that PARG inhibitors might indirectly compromise PARP function in the repair of DNA damage induced by certain agents [33,34].

In view of the scarcity of information available on the role of pADPr metabolism in trypanosomatids, our report about the existence of a PARG in *T. cruzi* as well as the possible role here reported for TcPARG in the cell cycle of these parasites contributes to this subject and other functional studies should be considered in order to fully understand the role of poly(ADP-ribosyl) ation in this parasites. Nevertheless, the most notable contribution achieved by the present work is given by the determination of the possible role that host cell pADPr metabolism could be playing in the cell infection process perpetrated by these parasites.

We have recently reported that PARP-1 silenced cells show lower susceptibility to *T. cruzi* infection and that a similar effect is observed in the presence of PARP inhibitors. However, in neither case the amastigote to trypomastigote differentiation process, or the amastigote intracellular replication seemed to be deeply affected, since trypomastigotes can be readily found in the supernatant of these cultures [14]. The results here presented suggest that PARG inhibitors could be affecting the cell cycle progression of this trypanosomatid and slowing down its growth, as observed in the epimastigote form. Nevertheless, the poly(ADP-ribose) glycohydrolase activity from the host cell seems to be playing a fundamental role during the process of infection. Our experiments, in which hPARG was inhibited with DEA or knocked down by iRNA, showed that the absence of this enzymatic activity has a deeper impact on *T. cruzi* infection than the absence of PARP activity. This result could potentially indicate that additional mechanisms other than those affected by PARP inhibition could be affected by PARG genetic or chemical ablation.

The absence of the PARG enzyme achieved by iRNA in A549 cells demonstrated a drastic reduction in the percentage of infected cells and in the number of amastigotes per cell when compared to wild type A549 infected cultures. However, a significant amount of trypomastigotes could still be detected in the supernatant of these cultures. In chemically PARG inhibited host cells, not only a significant reduction in the percentage of infected cells and in the number of amastigotes per cell was observed, but also a nearly complete absence of trypomastigotes in the culture supernatant was found, leading to a practically total abrogation of the infection process. The difference between these results highlights the crucial role of host cell PARG for the establishment and continuity of *T. cruzi* infection but also points out a possible role of the parasite enzyme; the implication of TcPARG in the intracellular amastigote replication and/or amastigote to trypomastigote differentiation processes cannot be ruled out.

Ba et al [35] reported that human cardiomyocytes produce reactive oxygen species (ROS) and inflammatory cytokines in response to *Trypanosoma cruzi*. Poly-ADPr formation and pADPrylation of mitochondrial membranes provide a secondary signal resulting in a positive feedback cycle of mitochondrial ROS production, DNA damage, and PARP-1/pADPr activation. ROS, either through direct modulation of cytosolic NF-κB or via PARP-1-dependent pADPr modification of p65-interacting nuclear proteins, contribute to cytokine gene expression and could be pointing out a link between ROS and inflammatory responses providing a clue to the pathomechanism of sustained inflammation in Chagas’ disease. Moreover, Pinto et al [36] show that the TNF/NF-kB axis participates in *T. cruzi* invasion of non-professional phagocytic epithelial cell lines, resulting in increased number of intracellular parasites, leading to the conclusion that NF-κB activation in non-immune cells elicited by paracrine factors released by immune cells represents a mechanism by which *T. cruzi* persists in the host. It has been recently published that phosphorylated cytoplasmic IκBα is diminished in shRNA PARG Lovo cells and consequently the intranuclear expression of NF-κB p65 and the total protein expression of the latter are decreased [37]. Al-Halabi et al. [38] have demonstrated that treatment with gallotannins inhibits NF-κB and slows the growth of human colon cancer xenografts. PARG inhibitor (gallotannin-GT) in A549 cells suppressed expressions of cytokines and chemokines blocking the activation of transcription factors NF-κB and activator protein-1 (AP-1) [39].

PARG activity in the host cell, because of its endo- and exo-glycosidase activity, could generate both free ADP-ribose or pADPr. Many evidences suggest that pADPr may exert different biological activities and a vast bibliography has been reported by Althaus et al. [40]. Apart from recruiting multiprotein complexes, pADPr may regulate the functions of specific protein domains, such as DNA binding or protein–protein interaction domains, catalytic activities, and post-translational modification sites. Moreover, pADPr size selectivity has been also demonstrated, thus adding more complexity to a pADPr-mediated regulatory mechanism. Hence, noncovalent interactions of pADPr with proteins are an important element of the intracellular-signaling network, which deserves attention. Blenn et al. [41] hypothesize that PARG could control Ca^2+^ shifts by converting pADPr into ADP-ribose, a possible activator of TRPM2 channels *in vitro*. Transient changes of intracellular calcium concentration could play a role in the invasion process and further studies have to be done to put insight to the signal mechanisms involved. We are carrying out experiments in order to better understand the mechanism of the invasion facilitated by inflammatory soluble factors and its relationship with pADPr metabolism.

Investigating the biological role of PARG enzyme has been challenging, since specific cell-permeable inhibitors are not available. Natural plant tannins like green tea polyphenols have been described, among other effects such as antioxidant activity, to inhibit PARG activity, with consequent accumulation of pADPr, and to induce growth inhibition and apoptosis in cancer cell lines [42–44]. Guida et al. [45] described the trypanocidal effects of compounds extracted from Green tea gallocatechin gallate and epigallocatechin gallate on the two clinically relevant forms of *T. cruzi*.

Recently PARG inhibitors has been suggested for alternative specific treatment for BRCA2-deficient and other HR-deficient tumors as single therapy [43], and the inhibitor GPI16552 has also been used previously in combination with the methylating agent temozolomide to reduce tumor cell growth, metastasis and prolong life in mice injected subcutaneously or intracranially with B16 melanoma cells [46]. This demonstrates that PARG inhibitors can be effectively used in an animal model without excessive systemic toxicity. Lately PARG is emerging as pharmacological target due to its low cellular abundance and new PARG inhibitors like modified salicylanilides [32] or rhodanine based compounds are being synthesized [31]. The aims in this field should be to develop new compounds that gather the desirable characteristics of ADP-HPD and DEA: the high specificity of the former inhibitor and the permeability of the latter.

The complexity of the relationship between PARP, PARG and cell invasion clearly deserves further investigation if PARP and PARG inhibitors are to be utilized to their fullest potential in *T. cruzi* infection.

## Supporting Information

Figure S1 Analysis of *Trypanosoma cruzi* poly(ADP-ribose) glycohydrolase expression over cell cycle. (A) Northern blot analysis of *Trypanosoma cruzi* CL Brener epimastigotes in synchronized cultures. Cultures with a density of 10^7^ parasites were incubated in the presence of hydroxyurea 15 mM for 20 hs. After this period, parasites were washed with PBS, resuspended in LIT medium and samples were drawn and analyzed by flow cytometry. Total RNA (10µg) from the parasites throughout the cell cycle was subjected to electrophoresis, transferred and hybridized with a radiolabeled probe corresponding to the full length of the coding region. rRNA was used as loading control. (B) Western blot analysis of TcPARG in *T. cruzi* epimastigotes. Protein extracts (35 µg) were electrophorezed and transferred to a nitrocellulose membrane and revealed with a 1:10000 dilution of polyclonal antibody against TcPARG followed by 1:6000 anti-mouse HRP conjugated antibody. β-tubulin was used as loading control. (C) Immunolocalization of TcPARG. Epimastigotes were fixed, treated with primary antibody (1:500) and Alexa Fluor 488 goat anti-mouse IgG antibody (1:600). Coverslips were washed with distilled water and mounted in Mowiol and then visualized using an Olympus BX41 microscope.

Figure S2 Sub-cellular localization of *Trypanosoma cruzi* poly(ADP-ribose) glycohydrolase. *T. cruzi* CL Brener transgenic epimastigotes carrying a copy of RED protein gene (A–D) or the RED-TcPARG fusion gene (E–H) in the pTREX expression vector were fixed for 25 min with 3.8% (W/V) formaldehyde in PBS at 4°C, mounted in Mowiol and visualized using an Olympus BX41 microscope. Cells were counterstained with DAPI to identify nuclear DNA and kinetoplastid (B,F). D and H show a merge between RED protein and DAPI signals. H, shows TcPARG and nuclear DNA colocalization. Bar: 10 µm.

Figure S3 Effect of PARG inhibitors on *T. cruzi* infection on Vero cells. The infection was allowed to proceed as described in Materials and Methods. In the PARG inhibited samples, DEA was kept in the growth medium at 1 µM throughout the experiment. At the indicated days, cells were fixed and stained by May Grünwald Giemsa technique. Cells were visualized using an Olympus BX41 microscope.

Figure S4 Effect of PARG inhibitors or PARG absence in the A549 host cell on *T. cruzi* infection. The infection was allowed to proceed as described in Materials and Methods. In the PARG inhibited samples, DEA was kept in the growth medium at 1 µM throughout the experiment. At day 6 post-infection cells were fixed and stained by May Grünwald Giemsa technique. Cells were visualized using an Olympus BX41 microscope.
