# Supplementary figures and images for: Host Cell Poly(ADP-Ribose) Glycohydrolase Is Crucial for Trypanosoma cruzi Infection Cycle
Source: PLoS One. 2013 Jun 12;8(6):e67356. doi: 10.1371/journal.pone.0067356 (PMC3680488; doi:10.1371/journal.pone.0067356)

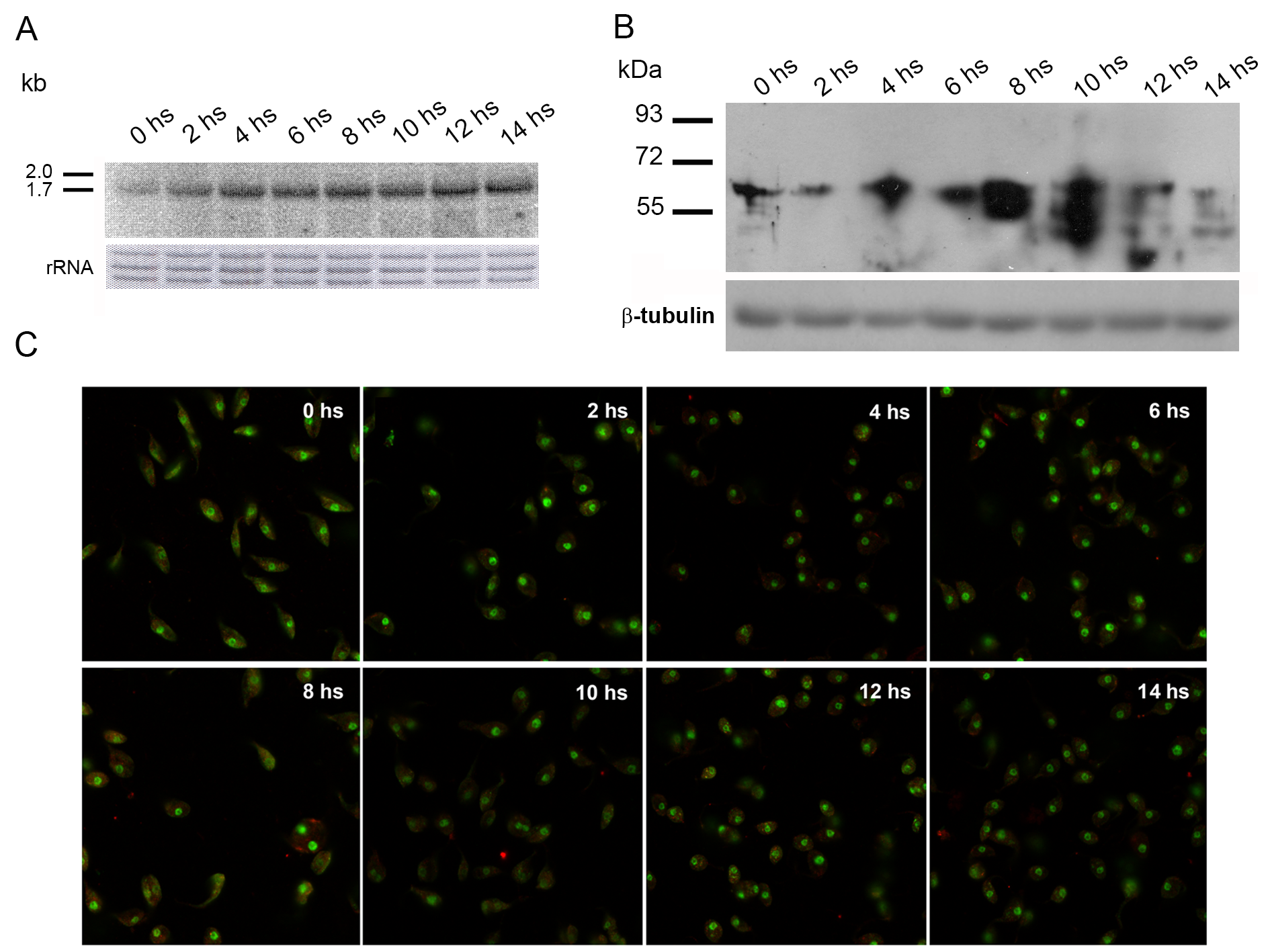

Supplement: Figure S1 — (A) Northern blot analysis of Trypanosoma cruzi CL Brener epimastigotes in synchronized cultures. Cultures with a density of 107 parasites were incubated in the presence of hydroxyurea 15 mM for 20 hs. After this period, parasites were washed with PBS, resuspended in LIT medium and samples were drawn and analyzed by flow cytometry. Total RNA (10µg) from the parasites throughout the cell cycle was subjected to electrophoresis, transferred and hybridized with a radiolabeled probe corresponding to the full length of the coding region. rRNA was used as loading control. (B) Western blot analysis of TcPARG in T. cruzi epimastigotes. Protein extracts (35 µg) were electrophorezed and transferred to a nitrocellulose membrane and revealed with a 1:10000 dilution of polyclonal antibody against TcPARG followed by 1:6000 anti-mouse HRP conjugated antibody. β-tubulin was used as loading control. (C) Immunolocalization of TcPARG. Epimastigotes were fixed, treated with primary antibody (1:500) and Alexa Fluor 488 goat anti-mouse IgG antibody (1:600). Coverslips were washed with distilled water and mounted in Mowiol and then visualized using an Olympus BX41 microscope. [file pone.0067356.s001.tif]

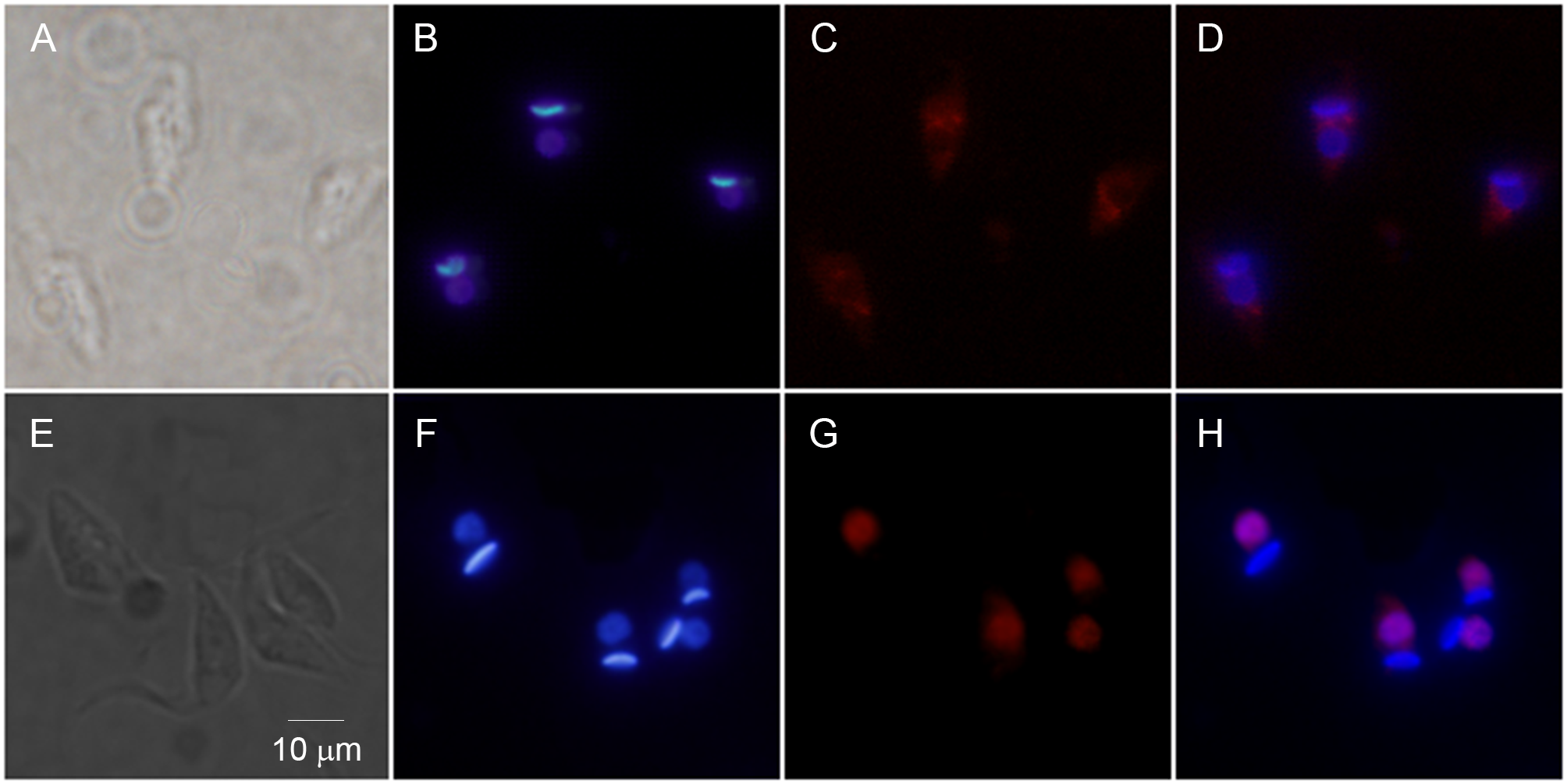

Supplement: Figure S2 — T. cruzi CL Brener transgenic epimastigotes carrying a copy of RED protein gene (A–D) or the RED-TcPARG fusion gene (E–H) in the pTREX expression vector were fixed for 25 min with 3.8% (W/V) formaldehyde in PBS at 4°C, mounted in Mowiol and visualized using an Olympus BX41 microscope. Cells were counterstained with DAPI to identify nuclear DNA and kinetoplastid (B,F). D and H show a merge between RED protein and DAPI signals. H, shows TcPARG and nuclear DNA colocalization. Bar: 10 µm. [file pone.0067356.s002.tif]

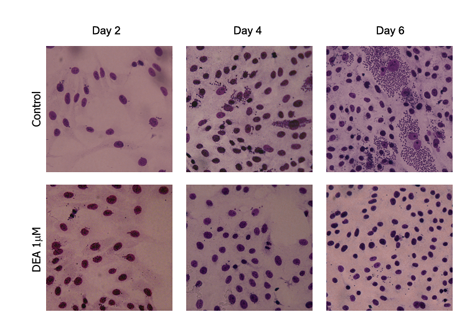

Supplement: Figure S3 — The infection was allowed to proceed as described in Materials and Methods. In the PARG inhibited samples, DEA was kept in the growth medium at 1 µM throughout the experiment. At the indicated days, cells were fixed and stained by May Grünwald Giemsa technique. Cells were visualized using an Olympus BX41 microscope. [file pone.0067356.s003.tif]

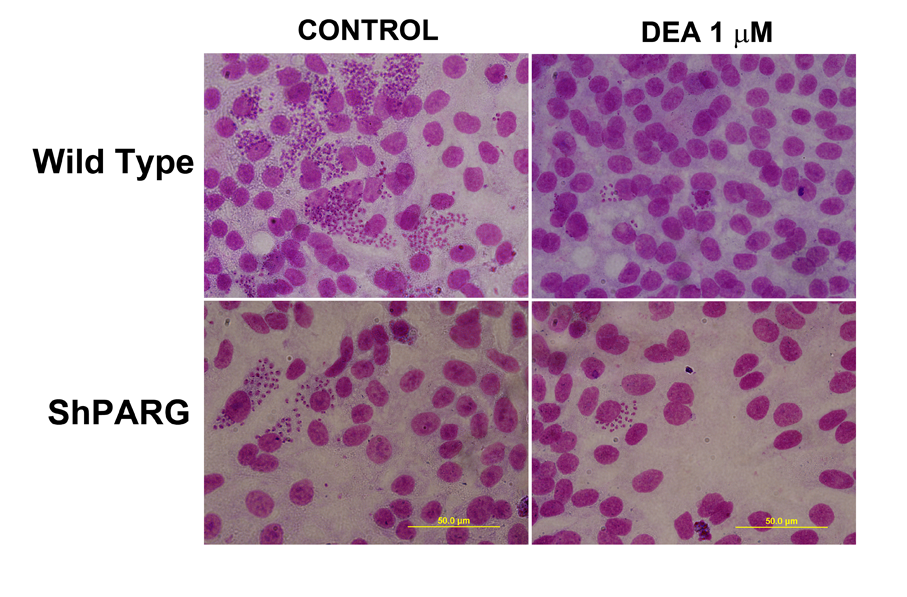

Supplement: Figure S4 — The infection was allowed to proceed as described in Materials and Methods. In the PARG inhibited samples, DEA was kept in the growth medium at 1 µM throughout the experiment. At day 6 post-infection cells were fixed and stained by May Grünwald Giemsa technique. Cells were visualized using an Olympus BX41 microscope. [file pone.0067356.s004.tif]
